# Environmentally Sustainable Functionalized WS_2_ Nanoparticles as Curing Promoters and Interface Modifiers in Epoxy Nanocomposites

**DOI:** 10.3390/nano15151145

**Published:** 2025-07-24

**Authors:** Lyazzat Tastanova, Amirbek Bekeshev, Sultan Nurlybay, Andrey Shcherbakov, Anton Mostovoy

**Affiliations:** 1 Oil and Gas Department, K. Zhubanov Aktobe Regional University, A. Moldagulova Ave. 34, Aktobe 030000, Kazakhstan; s.nurlybay@zhubanov.edu.kz; 2 Polymer Composites Laboratory, K. Zhubanov Aktobe Regional University, A. Moldagulova Ave. 34, Aktobe 030000, Kazakhstan; abekeshev@zhubanov.edu.kz; 3 Laboratory of Support and Maintenance of the Educational Process, Yuri Gagarin State Technical University of Saratov, Polytechnichskaya St. 77, 410054 Saratov, Russia; gassmed7@gmail.com; 4 Laboratory of Modern Methods of Functional Materials and Systems Research, Yuri Gagarin State Technical University of Saratov, Polytechnichskaya St. 77, 410054 Saratov, Russia; mostovoy19@rambler.ru

**Keywords:** epoxy resin, tungsten disulfide, functionalization, aminoacetic acid, nanocomposite, green nanofillers, modification, interfacial adhesion

## Abstract

This study investigates the effect of the surface functionalization of tungsten disulfide (WS_2_) nanoparticles with aminoacetic acid (glycine) on the structure, curing behavior, and mechanical performance of epoxy nanocomposites. Aminoacetic acid, as a non-toxic, bio-based modifier, enables a sustainable approach to producing more efficient nanofillers. Functionalization, as confirmed by FTIR, EDS, and XRD analyses, led to elevated surface polarity and greater chemical affinity between WS_2_ and the epoxy matrix, thereby promoting uniform nanoparticle dispersion. The strengthened interfacial bonding resulted in a notable decrease in the curing onset temperature—from 51 °C (for pristine WS_2_) to 43 °C—accompanied by an increase in polymerization enthalpy from 566 J/g to 639 J/g, which reflects more extensive crosslinking. The SEM examination of fracture surfaces revealed tortuous crack paths and localized plastic deformation zones, indicating superior fracture resistance. Mechanical testing showed marked improvements in flexural and tensile strength, modulus, and impact toughness at the optimal WS_2_ loading of 0.5 phr and a 7.5 wt% aminoacetic acid concentration. The surface-modified WS_2_ nanoparticles, which perform dual functions, not only reinforce interfacial adhesion and structural uniformity but also accelerate the curing process through chemical interaction with epoxy groups. These findings support the development of high-performance, environmentally sustainable epoxy nanocomposites utilizing amino acid-modified 2D nanofillers.

## 1. Introduction

Epoxy resins are applied in different industries, such as automotive, aerospace, marine, and electronics, due to their excellent mechanical characteristics, high thermal stability, and chemical resistance [1,2,3]. However, their inherent brittleness restricts the applicability of these materials in impact-prone or cyclic loading environments, where superior strength and enhanced fracture resistance are essential [4,5,6,7].

One promising strategy for optimizing mechanical and processing characteristics involves the incorporation of nanoscale modifiers, whose active surfaces are capable of strengthening interfacial bonding and influencing curing kinetics [8,9,10]. The high surface area and aspect ratio of the nanoparticles allow them to effectively suppress crack propagation, elevate stiffness, and facilitate strong interfacial bonding within polymer matrices even at low filler loadings [11,12,13,14]. For instance, nanoclays [1,3], potassium polytitanates [15,16], carbon nanotubes [17,18], and graphene derivatives [2,10] have been employed to augment interfacial compatibility and boost the mechanical performance of epoxy composites.

Recent advances highlight the effectiveness of functionalized 2D nanomaterials, although a direct comparison of modifiers shows significant differences in dispersion efficiency and interfacial bonding mechanisms [19,20,21,22]. Among the wide variety of nanofillers investigated to date, 2D transition metal dichalcogenides (TMDs), particularly tungsten disulfide (WS_2_), have emerged as promising reinforcing agents for polymer matrices [20,23,24]. WS_2_ possesses excellent thermal and chemical stability, high stiffness, and unique layered morphology, which can contribute to the mechanical strength, thermal conductivity, and tribological properties of polymer composites [25,26,27,28].

Despite the significant potential of tungsten disulfide as a reinforcing filler, its practical application is limited by its tendency to aggregate due to strong Van der Waals forces, low surface energy, and an intrinsically layered structure, all of which hinder uniform dispersion in organic matrices [29,30]. To address these drawbacks, surface functionalization techniques are used to optimize compatibility with epoxy resin and promote more homogeneous distribution.

Various strategies have been investigated to strengthen the interfacial coupling between WS_2_ and polymer matrices, including surface modification with silane coupling agents [31], polymer grafting [32], and treatment with bio-inspired modifiers [33,34]. Silane-based functionalization enables covalent attachment via Si–O–WS_2_ linkages, thereby enhancing both dispersion and interfacial adhesion. However, this method generally involves multistep synthesis and demands precise control over reaction conditions [31]. In contrast, grafting with conductive polymers introduces additional functionalities yet further increases the complexity of the synthetic process [32]. Tannic acid provides a simpler, eco-friendly alternative based on hydrogen bonding, though its thermal stability is limited [33]. Organophosphonate modification promotes filler–matrix compatibility through polar interactions but typically requires relatively high loading levels [34].

In pursuit of more sustainable and efficient functionalization routes, small biocompatible molecules containing both amino and carboxyl groups—capable of anchoring to the nanofiller surface and participating in matrix crosslinking reactions—are of particular interest [35]. Aminoacetic acid (glycine), the simplest amino acid, possesses both –NH_2_ and–COOH functional groups, allowing it to interact with inorganic nanoparticles as well as with reactive sites in epoxy systems [36,37,38]. Its availability, low toxicity, and environmental safety make glycine a promising candidate for WS_2_ surface modification. With its carboxyl group attaching to the nanoparticle surface and the amino group reacting with epoxy functionalities during curing, glycine not only enhances interfacial bonding but also improves the overall performance of the nanocomposite.

In previous studies, the authors successfully functionalized nanodiamonds [37] and aluminum nitride [38] with aminoacetic acid, which led to a significantly better nanoparticle dispersibility, strengthened interfacial compatibility, and superior mechanical performance of the resulting epoxy composites. Building on this foundation, the current study extends the application of this functionalization approach to a two-dimensional layered nanomaterial, tungsten disulfide (WS_2_). Due to WS_2_’s intrinsic layered structure, pronounced tendency to aggregate, and limited chemical reactivity, its surface modification requires additional adaptation and optimization.

This study builds upon prior work by adapting and experimentally validating an environmentally benign, one-step functionalization approach previously applied to 3D nanoparticles. The process avoids the use of aggressive chemical reagents and enables the stable covalent grafting of functional groups, thereby enhancing the compatibility of 2D-structured WS_2_ with the epoxy matrix. A comparative evaluation of composites containing unmodified and functionalized WS_2_ is carried out to comprehensively assess the efficiency of the proposed surface modification strategy.

The hypotheses of this study are as follows:

- The surface modification of WS_2_ with aminoacetic acid is expected to mitigate nanoparticle aggregation and facilitate uniform dispersion within the epoxy matrix;
- The introduced amino groups are anticipated to form covalent bonds with epoxy oligomers during curing, thereby strengthening interfacial adhesion;
- Collectively, these effects are projected to improve the mechanical properties and thermal stability of the nanocomposites relative to those containing unmodified WS_2_.

This study aims to explore how the functionalization of WS_2_ nanoparticles with aminoacetic acid influences their dispersion characteristics, interfacial interactions with the epoxy matrix, and the resulting structural, thermal, and mechanical behavior of the nanocomposites. To address this, the following objectives are pursued:

- To determine the optimum concentration of aminoacetic acid for effective surface modification of WS_2_;
- To analyze and compare the curing kinetics of epoxy systems containing unmodified and functionalized WS_2_;
- To evaluate the dispersion quality, microstructure, mechanical performance, and thermal stability of the resulting nanocomposites.

The findings are expected to contribute to the broader use of sustainable surface modification techniques and support the design of high-performance epoxy-based nanocomposites.

## 2. Materials and Methods

### 2.1. Materials

Diglycidyl ether of bisphenol A (DGEBA), industrial-grade ED-20 (CHIMEX Ltd., St. Petersburg, Russia), was applied as a polymer matrix. Curing was carried out using polyethylene polyamine (PEPA), an amine-type hardener produced by the same manufacturer. PEPA is a mixture of linear and branched aliphatic polyamines with many reactive –NH_2_ and –NH-groups, enabling efficient crosslinking at ambient temperature, which is advantageous for energy-saving in composite production.

Tris (2-chloroethyl) phosphate (TCEP), supplied by Xuancheng City Trooyawn Refined Chemical Industry Co. (Xuancheng, China) with a reported purity of 95–99%, was used as a reactive plasticizer and flame retardant. TCEP, being a phosphorus-containing compound, enhances the flame retardancy of the polymer system, lowers the viscosity of the epoxy resin, and facilitates effective wetting and uniform dispersion of the filler. Its plasticizing action also has a positive effect on the strength and processing performance of the cured material. The presence of both polar and nonpolar functional groups in the TCEP molecule promotes its efficient incorporation into the epoxy network.

Tungsten disulfide nanoparticles (WS_2_), characterized by a layered structure, high aspect ratio, lubricity, and thermal stability, were used as a nanostructuring additive. WS_2_ powder with a purity of 99.9% was produced by Simplex Ltd. (Perm, Russia). To achieve better compatibility with the epoxy matrix, nanoparticles were functionalized with aminoacetic acid (glycine) obtained from Vita Reactive Ltd. (Dzerzhinsk, Russia).

### 2.2. Functionalization of WS_2_ Nanoparticles

The functionalization was carried out as follows: 0.5 g of WS_2_ powder was dispersed in 100 mL of aqueous aminoacetic acid solution with concentrations of 2.5, 5, 7.5, and 10 wt%. The dispersion was carried out using an ultrasonic homogenizer for 15 min until a homogeneous suspension was obtained. The mixture was then subjected to reflux heating at 80 °C for 12 h with constant mechanical stirring (100 rpm). Upon the completion of the reaction, the suspension was centrifuged, the solid phase was washed twice with distilled water to remove excess aminoacetic acid, and then it was dried in a convection drying oven at 80 °C for 5 h.

### 2.3. Preparation of Nanocomposites

The base composition included 100 parts of ED-20, 40 parts of TCEP, and 15 parts of PEPA. Functionalized WS_2_ nanoparticles were added in amounts of 0.01; 0.05; 0.1; 0.25; 0.5; 0.75; 1.0; 2.5; and 5 parts per hundred resin (phr).

To ensure uniform distribution and prevent agglomeration, the nanoparticles were premixed with the epoxy resin and then treated with ultrasound (400 W, 22 ± 2 kHz) for 60 min at <50 °C using an external cooling system. This step is critical to form a well-developed interfacial zone and promote more a homogeneous structure of the composite.

After ultrasonication, the mixture was degassed under vacuum (−0.09 MPa, 25 ± 5 °C) for 30 min. The PEPA hardener was then introduced, and the composition was stirred for 5 min with gentle manual stirring. Curing was carried out at room temperature (20–25 °C) for 24 h; after that, the samples were additionally incubated at 80 °C for 2 h to raise the degree of crosslinking and elevate thermal stability.

### 2.4. Methods of Analysis

SEM: The fracture morphology and distribution of nanoparticles in the epoxy composite structure were studied using TESCAN VEGA 3 SBH SEM (Brno, Czech Republic). The samples were pre-metalized with a gold layer to prevent charge accumulation on the surface.

BET: Specific surface area and porosity were determined by BET on a Quantachrome NOVA 2200e analyzer (Boynton Beach, FL, USA) using nitrogen adsorption/desorption isotherms. Prior to measurement, samples were degassed at 150 °C for ~3 h until constant mass was reached. Measurements were performed at liquid nitrogen temperature (~78 K) in the relative pressure range of 0.03–0.3 P/P_0_. The BJH model was used to calculate the pore size distribution.

DSC: Curing kinetics was studied by the differential scanning calorimetry method on a DTAS 1300 device (Samara, Russia) using aluminum cells. The mass of the samples was 20 mg.

Mechanical tests were performed in accordance with ISO standards on a universal testing machine at a temperature of 23 ± 2 °C and relative humidity of 50 ± 5%. The test conditions for the physical and mechanical properties of the composites are summarized in Table 1.

Thermogravimetric analysis (TGA) of composites was performed on a Q-1500D derivatograph (MOM, Budapest, Hungary) in the temperature range of 20–800 °C with a linear heating rate of 10 °C/min in a nitrogen atmosphere. The mass of the sample was 100 mg. The onset temperature of thermal decomposition (T_o_) and the temperature of maximum decomposition rate (T_max_) were recorded.

Thermal conductivity and thermal resistance were measured using an ITP-MG4 “100” instrument (Stroypribor, Chelyabinsk, Russia) according to ISO 22007-2:2015 [45]. Rectangular specimens of the size 10 × 10 × 2 mm^3^ were tested at steady-state heat flux.

FTIR spectra were recorded using an IRTracer-100 spectrometer (Shimadzu, Kyoto, Japan) in the range of 400–4000 cm^−1^ with a resolution of 4 cm^−1^. The fluctuation intensity of the IR spectra was determined using Shimadzu’s “Lab Solutions IR” software (version 1.21). The method was used to confirm chemical modification of nanoparticles.

X-ray diffraction (XRD): Phase analysis was performed on an ARL X’TRA diffractometer (Thermo Fisher Scientific, Waltham, MA, USA) using Cu Kα radiation (λ = 1.5406 Å). Scanning conditions were the following: range 2θ = 5–80°, scanning step—0.02°, speed—1°/min, voltage—40 kV, current—30 mA. The phase composition was determined using a PDF database of International Centre for Diffraction Data (ICDD). WS_2_ (PDF#030-65-7515) [46] and aminoacetic acid (C_2_H_5_NO_2_, PDF#000-01-0865) [47] phases were identified.

## 3. Results and Discussion

### 3.1. Wettability and Interfacial Compatibility

Interfacial compatibility, which governs the structural integrity and performance of polymer composites, is a key factor when integrating inorganic fillers into organic matrices [48,49]. In this study, the effect of aminoacetic acid surface functionalization on the wettability of tungsten disulfide nanoparticles with plasticized epoxy resin was assessed via contact angle measurements.

**Figure 1:**
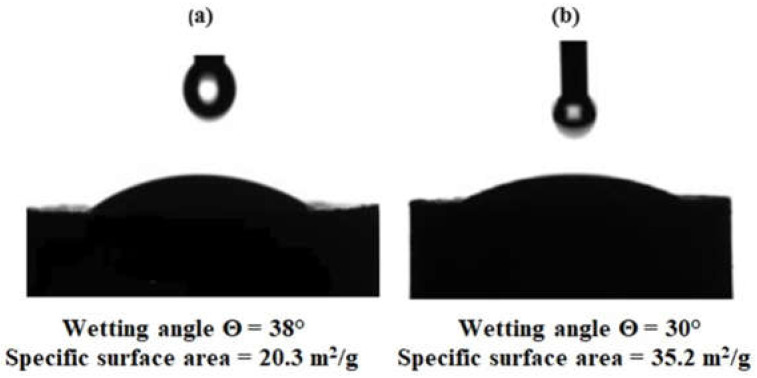
Wettability characteristics of WS_2_ nanoparticles in plasticized epoxy resin (ED-20 + TCEP): (**a**) untreated WS_2_; (**b**) WS_2_ after functionalization with aminoacetic acid.

The unmodified WS_2_ exhibited a contact angle of 38° (Figure 1a), indicating relatively poor wettability compared to the functionalized WS_2_ (30°) (Figure 1b) and the highly polar epoxy matrix. This is attributed to the lack of polar functional groups on the pristine WS_2_ surface, which hinders optimal interfacial interactions. Functionalization with aminoacetic acid markedly enhanced wettability, as indicated by the reduced contact angle, which in turn facilitated better nanoparticle dispersion and interfacial adhesion.

This is attributed to the presence of polar functional groups (–NH_2_ and –COOH), which elevate the surface energy. The specific surface area of WS_2_ rose from 20.3 to 35.2 m^2^/g, further facilitating interfacial interaction.

The results demonstrate that amino acid functionalization is an effective strategy for strengthening the interface between WS_2_ and epoxy matrices.

### 3.2. Confirmation of Functionalization

The successful chemical modification of WS_2_ surface with aminoacetic acid was confirmed by FTIR spectroscopy, EDS, and X-ray diffraction methods. These techniques were applied to verify the covalent incorporation of organic moieties onto the filler surface, assess changes in chemical bonding, and identify alterations in crystalline structure that are associated with surface modification.

**Figure 2:**
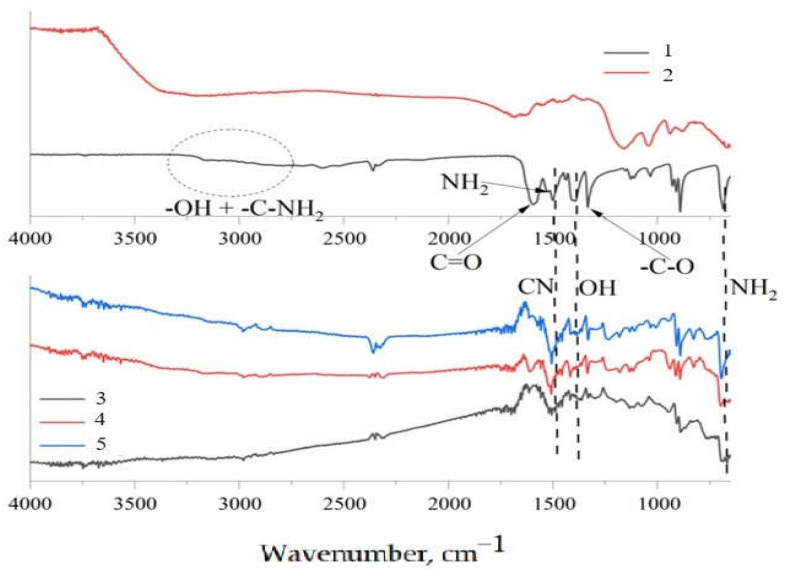
FTIR spectra of (1) aminoacetic acid, (2) pristine WS_2_, (3) 5% aminoacetic acid-modified WS_2_, (4) 7.5% aminoacetic acid-modified WS_2_, and (5) 10% aminoacetic acid-modified WS_2_.

In the IR spectra of the modified particles (Figure 2), a decrease in the intensity of the –OH band (3400 cm^−1^) was observed, indicating the participation of WS_2_ hydroxyl groups in condensation or esterification reactions with the carboxyl groups of aminoacetic acid. At the same time, absorption bands characteristic of aminoacetic acid–C = O (~1700 cm^−1^) and C–N (~1250 cm^−1^) appeared, stable even after washing, thereby confirming the presence of non-hydrolyzable bonds. The absorption at ~1700 cm^−1^ corresponds to the carbonyl (C = O) stretch of ester groups, while the band near 1250 cm^−1^ indicates the formation of C–N bonds, both of which are signatures of covalent grafting onto the WS_2_ surface. The intensity of these bands rose with increasing modifier concentration, demonstrating the concentration-dependent grafting efficiency and confirming the controllability of the grafting process. These spectral changes, as presented in Figure 2, provide molecular-level evidence of successful chemical interaction between the modifier and the WS_2_ surface.

**Figure 3:**
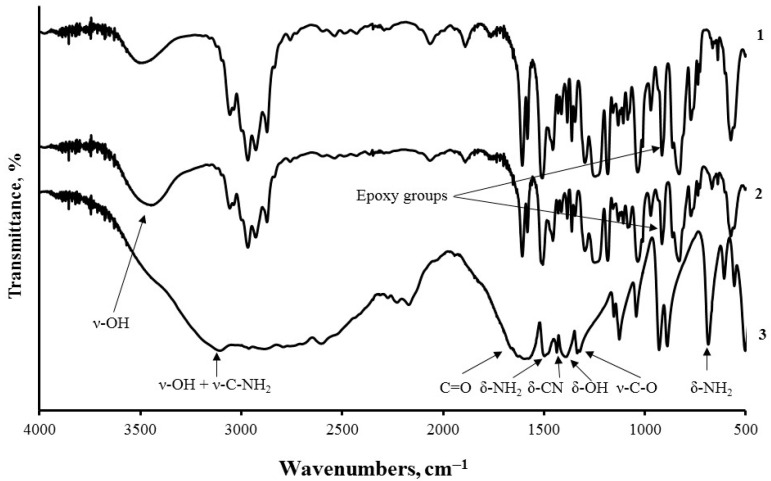
FTIR spectra of (1) neat ED-20 epoxy resin, (2) mixture of epoxy resin with aminoacetic acid, and (3) pure aminoacetic acid.

FTIR analysis of the system ED-20—aminoacetic acid (Figure 3) made it possible to understand the crosslinking mechanism. When aminoacetic acid was introduced into the epoxy resin, a 38% reduction in the intensity of the absorption band at 910 cm^−1^—attributed to the epoxy ring—was observed, confirming the reaction between its amine group (–NH_2_) and the epoxy moieties. At the same time, a 22% increase in the hydroxyl group band at 3470 cm^−1^, indicative of secondary –OH group formation during epoxy ring opening, was detected. Together, these spectral changes provide clear evidence of covalent bonding between aminoacetic acid and the epoxy matrix—an essential factor in the formation of a crosslinked polymer network.

**Figure 4:**
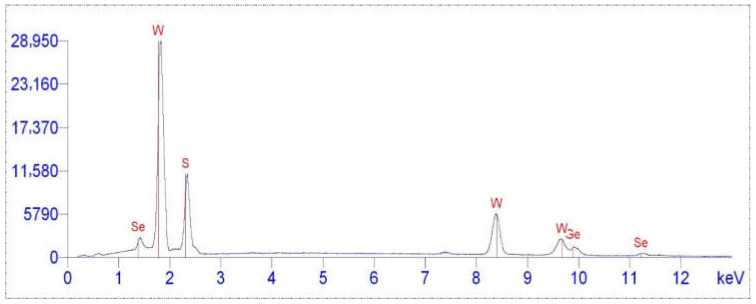
EDS spectrum of WS_2_ nanoparticles.

**Figure 5:**
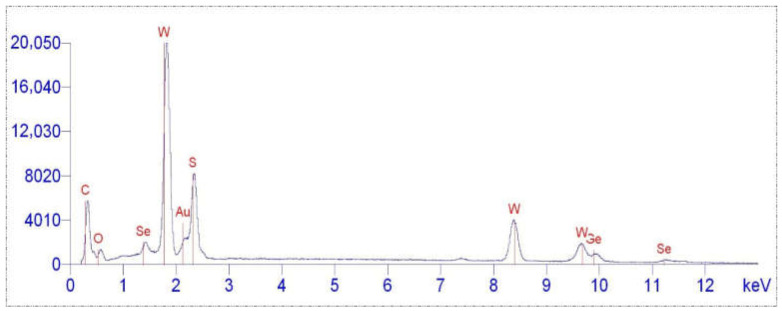
EDS spectrum of WS_2_ nanoparticles functionalized with aminoacetic acid.

Energy-dispersive X-ray Spectroscopy (EDS) spectra (Figure 4 and Figure 5) demonstrate stable content of W and S before and after the functionalization of the tungsten disulfide nanoparticle surface with aminoacetic acid, as well as the appearance of C and O signals after modification, indicating the anchoring of organic aminoacetic acid groups. The stability of W and S content, alongside the appearance of organic signals, suggests that the surface modification adds chemically distinct organic layer without any disruption of the inorganic core.

**Figure 6:**
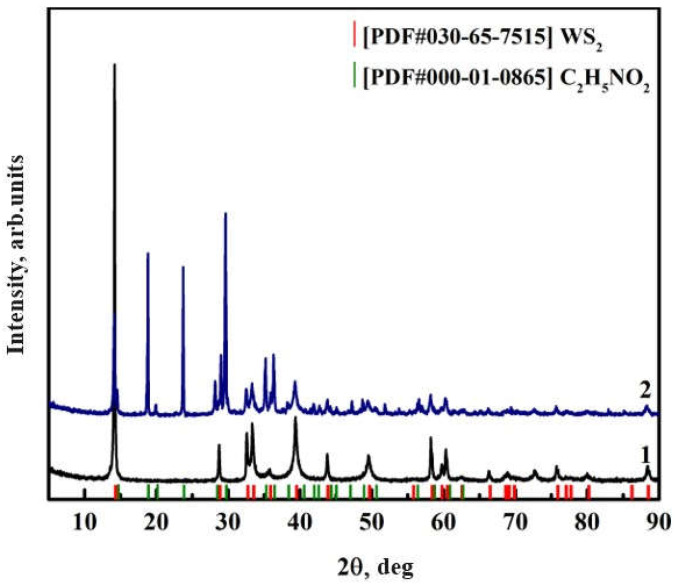
XRD patterns of (1) pristine WS_2_ nanoparticles and (2) WS_2_ nanoparticles treated with aminoacetic acid.

X-ray diffraction (XRD) analysis showed (Figure 6) that the main diffraction peaks of WS_2_ (PDF#030-65-7515) are kept after functionalization, confirming the preservation of the crystalline structure [34,46]. However, the decrease in peak intensity and broadening of reflections indicate partial exfoliation of the layered structure and disordering of the outer layers, likely due to the introduction of organic grafts. Weak diffraction features consistent with aminoacetic acid (PDF#000-01-0865) were also observed in the 10–30° region, supporting the presence of surface-bond organic fragments [36,47].

In addition to peak broadening and intensity reduction, slight shifts in the diffraction peak positions were observed upon functionalization. These changes can be attributed to localized surface strain induced by the covalent grafting of aminoacetic acid, including the formation of ester bonds (C–O–W) and the presence of polar functional groups (–NH_2_ and –COOH). Such modifications may alter the electron density distribution and slightly distort the lattice parameters near the nanoparticle surface. Furthermore, the ultrasonic-assisted treatment may cause partial exfoliation and the disruption of layer stacking, contributing both to peak broadening and minor angular shifts.

Despite these surface-level alterations, the retention of the primary diffraction pattern confirms the preservation of the crystalline WS_2_ core. These XRD observations are in agreement with the FTIR and EDS data and collectively confirm the successful surface modification of WS_2_ without the formation of secondary crystalline phases.

**Figure 7:**
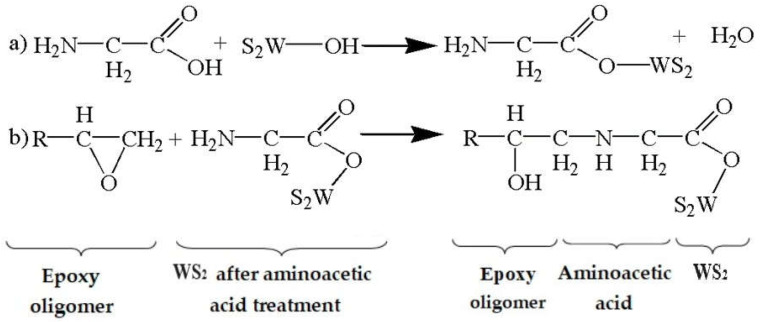
Proposed reaction mechanism for the interaction of WS_2_ with aminoacetic acid and epoxy oligomer: (**a**) esterification of hydroxylated WS_2_ surface with aminoacetic acid; (**b**) ring-opening reaction of epoxy group with the amino-functionalized WS_2_ surface.

The proposed mechanism of WS_2_ functionalization with aminoacetic acid involves a two-step pathway (Figure 7). In the first stage, the carboxyl groups (–COOH) of aminoacetic acid react with hydroxyl groups (–OH) naturally present on the WS_2_ surface due to partial hydrolysis or oxidation of sulfur atoms. As a result, stable ester bonds (C–O–W) are formed, covalently anchoring the organic modifier to the nanoparticle surface (Figure 7a). This esterification is facilitated by mild heating and ultrasonic treatment, which together advance the molecular-level contact between the reactants. Importantly, this approach ensures that the aminoacetic acid is not merely physically absorbed but chemically grafted, leading to a more stable and functionally active interface.

In the second stage, the remaining amino groups (–NH_2_) on the grafted aminoacetic acid become available to react with the epoxy resin during curing. These groups open the epoxy rings and form new covalent bonds, chemically connecting the nanoparticle to the polymer network (Figure 7b). As a result, the WS_2_ particles are not merely physically embedded in the matrix but are chemically integrated into the crosslinked network, thereby strengthening interfacial adhesion and contributing to a more uniform and robust composite.

While esterification and amine–epoxy coupling are the predominant reaction pathways, additional interactions may also occur. These include hydrogen bonding between residual –NH_2_, –COOH, or –OH groups and polar segments of the epoxy matrix, as well as the physical sorption of non-grafted aminoacetic acid. Although the latter can contribute to transient interactions, it is largely removed by washing. Spectral analyses (FTIR, EDS) indicate that covalent bonding is the dominant mechanism, ensuring that only firmly grafted organic moieties remain on the WS_2_ surface.

This functionalization mechanism can be viewed as the construction of a molecular bridge between two chemically dissimilar components—WS_2_ and the epoxy matrix. Acting as a bifunctional linker, aminoacetic acid enables the covalent anchoring of its carboxyl group (–COOH) to the hydroxylated WS_2_ surface while exposing its amino group (–NH_2_) for subsequent reaction with epoxy groups during curing. This dual reactivity not only promotes nanoparticle dispersion but also ensures their chemical integration into the crosslinked polymer network. As a result, surface-modified WS_2_ nanoparticles differ fundamentally from inert fillers: they contribute to both the structural and chemical architecture of the final composite.

### 3.3. Curing Thermokinetics of Epoxy Composites

The introduction of functionalized WS_2_ nanoparticles into the ED-20 epoxy system significantly influenced the curing kinetics. The enlarged specific surface area and the presence of reactive groups on the modified particle surface promote stronger interaction with the matrix, thereby accelerating gelation and crosslinking and increasing the homogeneity of the polymer network.

**Figure 8:**
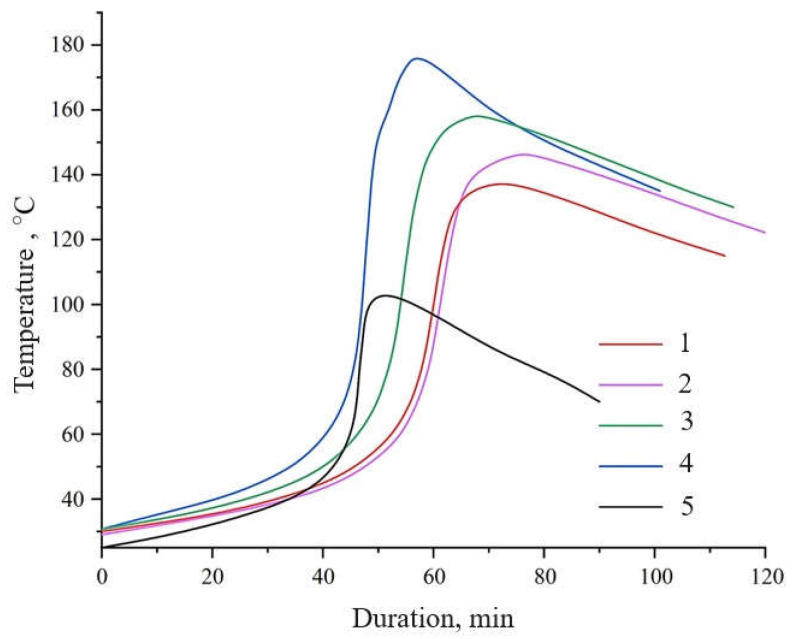
Temperature evolution during the curing of epoxy composites containing pristine and aminoacetic acid-functionalized WS_2_ nanoparticles. Curves: (1) unmodified WS_2_; (2) WS_2_ functionalized with 5 wt% aminoacetic acid; (3) WS_2_ functionalized with 7.5 wt% aminoacetic acid; (4) WS_2_ functionalized with 10 wt% aminoacetic acid; (5) epoxy matrix without WS_2_.

The temperature–time profiles of the curing epoxy composites (Figure 8) showed that the addition of untreated WS_2_ prolonged the gelation time (from 45 to 51 min) and extended the total curing duration (from 53 to 71 min) while simultaneously raising the maximum self-heating temperature (T_max_) to 138 °C (curve 1). This effect is attributed to the thermal conductivity of WS_2_ and its barrier properties, which hinder the diffusion of the hardener.

Functionalization of WS_2_ with aminoacetic acid significantly altered the curing behavior of the system (curves 2–4). As the concentration of aminoacetic acid used for WS_2_ surface modification increased from 5 to 10 wt%, T_max_ rose to 147–175 °C, while gelation and curing times shortened from 49 to 38 min and from 75 to 57 min, respectively (Table 2). These changes reflect stronger chemical interactions at the filler–matrix interface, which indirectly facilitate the formation of a crosslinked network.

**Figure 9:**
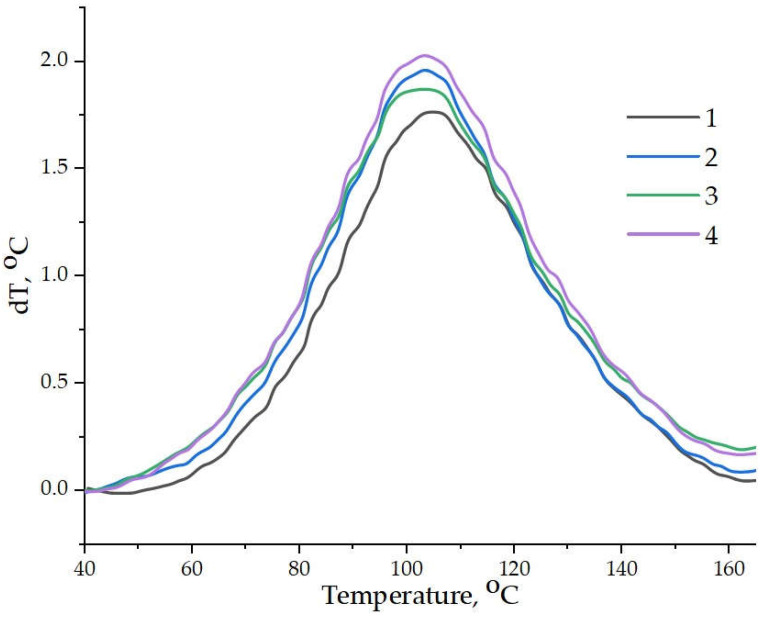
DSC curves of epoxy compositions containing pristine and aminoacetic acid-functionalized WS_2_ nanoparticles: (1) ED-20 + TCEP + WS_2_ + PEPA; (2) ED-20 + TCEP + WS_2_ (2.5 wt% aminoacetic acid) + PEPA; (3) ED-20 + TCEP + WS_2_ (5.0 wt% aminoacetic acid) + PEPA; (4) ED-20 + TCEP + WS_2_ (7.5 wt% aminoacetic acid) + PEPA.

Differential scanning calorimetry (DSC) data (Figure 9, Table 3) supported the findings obtained from the temperature–time curing profiles. The incorporation of amino-functionalized WS_2_ nanoparticles reduced the onset temperature of curing from 51 °C to 43 °C, indicating that crosslinking began earlier. Additionally, the overall reaction enthalpy rose, indicating the more complete and efficient formation of the crosslinked network. The peak heat release temperature (T_max_ ≈ 102 °C) remained nearly unchanged, suggesting that the effect of functionalization is primarily associated with the initiation stage and the total reaction enthalpy.

Consequently, the functionalized WS_2_ nanoparticles perform a dual role, simultaneously acting as mechanical reinforcers that enhance stiffness and fracture toughness, and as chemical accelerators that promote curing by introducing active sites for epoxy crosslinking. This synergistic behavior leads to faster gelation and enhanced uniformity of the crosslinked network, as demonstrated by thermokinetic and mechanical data.

### 3.4. Fracture Morphology and Mechanical Properties

**Figure 10:**
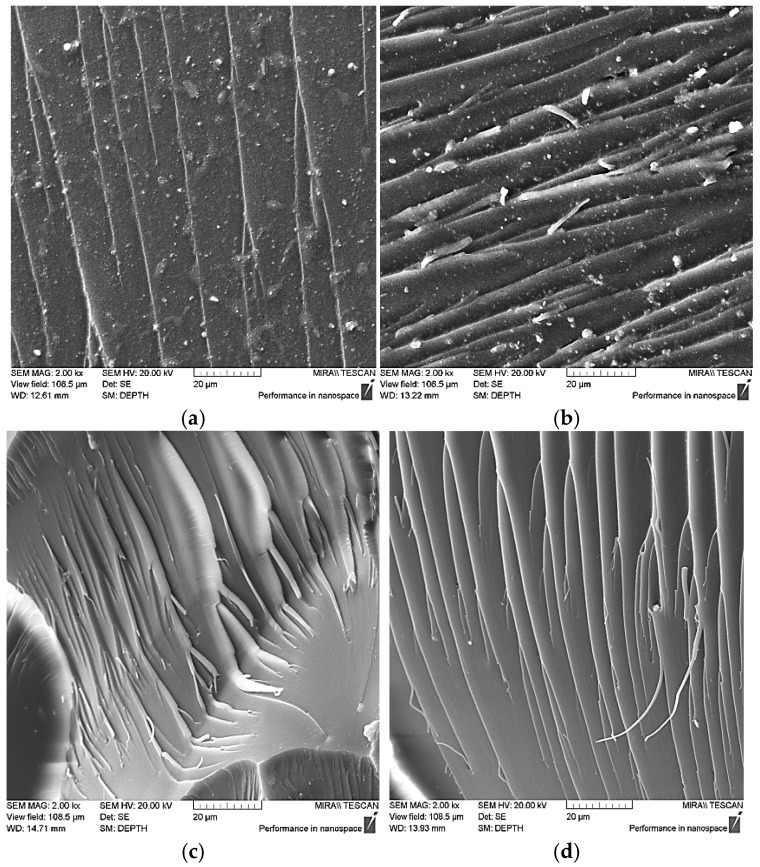
SEM images of fracture surfaces of epoxy composites: (**a**) neat epoxy composition (ED-20 + TCEP + PEPA); (**b**) with pristine WS_2_ nanoparticles; (**c**) containing WS_2_ nanoparticles functionalized with 2.5 wt% aminoacetic acid; (**d**) containing WS_2_ nanoparticles functionalized with 5.0 wt% aminoacetic acid.

SEM analysis revealed pronounced differences in fracture morphology depending on the type of filler used (Figure 10). The neat epoxy matrix exhibited a smooth fracture surface with straight, propagating microcracks (Figure 10a), indicative of brittle failure, low fracture energy, and minimal plastic deformation [37,50]. The incorporation of unmodified WS_2_ nanoparticles resulted in a more heterogeneous fracture profile (Figure 10b), characterized by crack branching, surface delamination, and deeper surface irregularities, suggesting increased fracture energy and partial plastic deformation. In contrast, composites reinforced with functionalized WS_2_ (Figure 10c,d) displayed tortuous crack paths, fibrillar structures, and river-like patterns—features typically associated with enhanced fracture resistance. These morphological changes point to stronger interfacial bonding and more effective stress transfer within the composite system.

Mechanical testing confirmed the reinforcing effect of WS_2_ nanoparticles (Table 4). At an optimal loading of 0.5 phr, the incorporation of pristine WS_2_ resulted in more than a 2.5-fold increase in flexural strength and a 2.3-fold rise in elasticity modulus relative to the neat epoxy. This simultaneous enhancement is likely attributed to improved interfacial load transfer and the ability of the filler–matrix interface to redirect or arrest propagating cracks. However, at filler contents exceeding 0.5 phr, mechanical performance declined, primarily due to particle agglomeration and the formation of local stress concentrations.

The functionalization of WS_2_ with aminoacetic acid amplified the positive effect: at 0.5 phr, flexural strength reached 168 MPa, modulus—5282 MPa, tensile strength—82 MPa, impact strength—17.0 kJ/m^2^. This is explained by the formation of strong covalent bonds at the interface and the participation of modified particles in the crosslinking process. Thus, functionalized nanoparticles perform a dual role: they reinforce the matrix mechanically and promote the formation of a dense mesh due to chemical interaction with the epoxy resin.

To evaluate the reliability of the observed differences, statistical analysis was conducted using a 95% confidence interval (n = 5, t = 2.776). Confidence intervals (CIs) and p-values were calculated for selected composite formulations. The results indicate that surface modification with aminoacetic acid yields statistically significant improvements in both flexural and tensile strength. Although the enhancement in impact toughness is less significant from a statistical standpoint, it remains a meaningful and practically relevant outcome.

### 3.5. Optimization of WS_2_ Concentration and Aminoacetic Acid Functionalization Level

**Figure 11:**
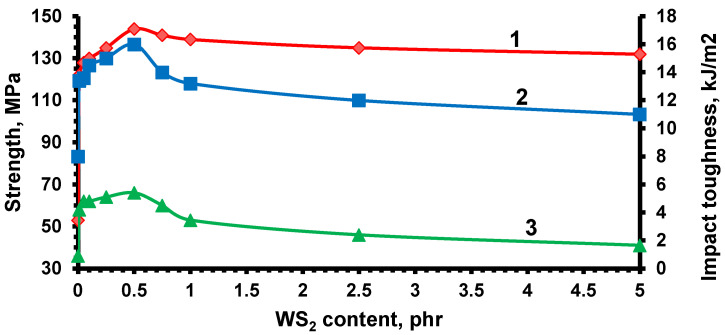
Effect of WS_2_ content on mechanical properties of epoxy nanocomposites: 1—flexural strength, 2—impact toughness, 3—tensile strength.

The systematic variation of WS_2_ content in the range of 0.01–5.0 phr enabled the identification of an optimal loading level for reinforcing the epoxy matrix. In all mechanical tests (Figure 11), a consistent improvement in performance was observed with increasing nanoparticle concentration, reaching a maximum at 0.5 phr. Beyond this threshold, properties deteriorated due to agglomeration, structural inhomogeneity, and the formation of localized stress zones. Consequently, 0.5 phr was established as the optimal nanofiller concentration, providing the best compromise between tensile strength, stiffness, and impact resistance while maintaining good dispersion stability.

**Figure 12:**
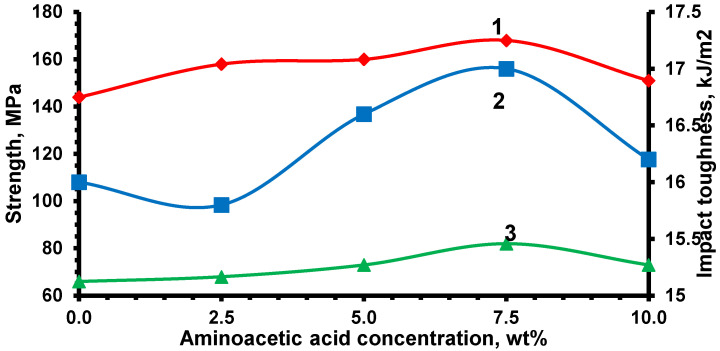
Effect of aminoacetic acid concentration used for WS_2_ surface functionalization on mechanical properties of epoxy nanocomposites containing 0.5 phr WS_2_: 1—flexural strength, 2—impact toughness, 3—tensile strength.

The effect of aminoacetic acid concentration (2.5–10 wt%) used for WS_2_ surface functionalization was distinctly nonlinear (Figure 12). The most pronounced improvement in all mechanical parameters was observed at 7.5 wt%, which corresponds to the highest interfacial bonding efficiency. This can be attributed to the optimal density of amine and carboxyl groups on the filler surface, enabling the formation of both covalent and hydrogen bonds with the epoxy matrix. Further increase in modifier content led to a slight decline in performance, likely due to nanoparticle agglomeration and the oversaturation of the interfacial surface with reactive groups, which may cause local overconsolidation and restrict the flexibility of the crosslinked network [51,52,53]. Accordingly, the optimal formulation was determined to be 0.5 phr WS_2_ functionalized with 7.5 wt% aminoacetic acid.

Composites containing the optimal WS_2_ content (0.5 phr) demonstrated consistent improvements across all investigated properties compared to the neat epoxy matrix. However, surface functionalization of nanoparticles with 7.5 wt% aminoacetic acid led to a statistically significant increase in strength characteristics—boosting flexural strength by 16.7% and tensile strength by 24.2%—as a result of strengthened interfacial adhesion and a more uniform distribution of WS_2_ within the matrix. In contrast, its effect on impact toughness (an increase of 6.25%) is statistically less significant, indicating the dominant role of the WS_2_ nanoparticles themselves in crack retardation mechanisms under dynamic loading. This confirms that functionalization is critical for maximizing static strength but secondary for impact toughness, where the filler plays the primary role.

The aminoacetic acid functionalization strategy employed in this work demonstrates several advantages over previously reported WS_2_ modification methods. These differences are summarized in Table 5, which compares key characteristics such as interaction mechanisms, processing conditions, environmental considerations, and epoxy compatibility. The comparison highlights the unique dual reactivity and environmentally sustainable character of the proposed method, which enables both covalent grafting and curing promotion.

## 4. Conclusions

This study demonstrates that the functionalization of tungsten disulfide nanoparticles with aminoacetic acid provides an effective strategy for simultaneously reinforcing interfacial interactions with the epoxy matrix, accelerating curing kinetics, and enhancing the mechanical performance of the resulting nanocomposites.

The surface modification of WS_2_ is accompanied by the appearance of polar functional groups (–NH_2_, –COOH) as confirmed by FTIR, EDS, and XRD analyses. This leads to a decrease in the contact angle, an increase in the specific surface area, and the formation of a chemically active interface. The functionalized particles promote stronger interaction with the matrix, indirectly manifested in accelerated curing, as evidenced by a reduced reaction onset temperature and increased polymerization enthalpy.

Morphological analysis revealed that functionalized WS_2_ nanoparticles contribute to the formation of energy-dissipating microstructures, including fibrillar textures and tortuous crack paths, which are indicative of enhanced resistance to crack propagation and more effective stress transfer across the filler–matrix interface.

Mechanical tests demonstrated a substantial enhancement in strength, stiffness, and impact toughness at the optimal WS_2_ content of 0.5 phr. When functionalized with 7.5 wt% aminoacetic acid, mechanical performance increased by 170–190% compared to the neat epoxy, confirming the synergistic effect of surface modification and nanostructural reinforcement. However, exceeding 0.5 phr WS_2_ or using aminoacetic acid concentrations above 7.5 wt% led to a decline in properties, attributed to nanoparticle agglomeration and the disruption of the interfacial architecture.

Compared to other reported functionalization strategies for WS_2_, this approach combines dual-site chemical reactivity with aqueous processing and the use of non-toxic reagents, thereby offering advantages in both composite performance and environmental sustainability.

The observed gains in mechanical performance underscore the potential of the developed nanocomposites for advanced engineering applications that demand high strength, toughness, and reliability. These nanocomposites may be of interest for industries such as aerospace, automotive, and electronics, where lightweight materials with high mechanical strength, thermal stability, and rapid processing are in demand. Stronger interfacial bonding and accelerated curing kinetics demonstrated in this study can contribute to the development of more energy-efficient and durable structural components. However, the industrial application of such nanomaterials will require addressing several practical challenges, including ensuring uniform nanoparticle dispersion in large-volume systems, maintaining consistency during composite fabrication, and complying with environmental and safety regulations. The further optimization of surface chemistry, processing conditions, and lifecycle performance will be essential for transitioning this approach from laboratory to real-world use.

While the study was conducted under well-controlled and practically relevant laboratory conditions, certain aspects remain beyond its current scope. In particular, long-term performance parameters such as aging resistance, environmental durability, and chemical stability under operational stressors were not assessed. Additionally, the environmental and economic feasibility of scaling up the functionalization process requires further evaluation. To facilitate the practical implementation of the developed nanocomposites, future research should address their long-term reliability, processing compatibility, and performance stability under conditions representative of industrial use. Despite these limitations, the results obtained provide a solid foundation for advancing environmentally friendly strategies in nanocomposite design.

Thus, the amino acid-modified WS_2_ nanoparticles simultaneously accelerate curing through chemical interactions and reinforce the matrix by strengthening interfacial bonding.

## Figures and Tables

**Table 1:** Methods and conditions of mechanical tests of developed epoxy composites.

Property	Methodology	Test Conditions
Flexural strength and modulus	ISO 178:2019 [39]	Crosshead speed 50 mm/min
Tensile strength and modulus	ISO 527-2:2012 [40]	Dog bone specimens, 50 mm gauge, speed 5 mm/min
Compressive strength	ISO 604:2002 [41]	Cylinder specimens, speed 1 mm/min
Impact strength	ISO 179-1:2010 [42]	Charpy method, uncut specimens
Brinell hardness	ISO 2039-1:2001 [43]	10 mm indenter, load 500 N
Vicat softening temperature	ISO 306:2022 [44]	Load 50 N, heating rate 50 °C/h

**Table 2:** Curing parameters of epoxy composites containing pristine and functionalized WS_2_.

Epoxy Composition ^1^	Gelation Time, Min	Curing Time, Min	T_max_^2^, °C
Control (ED-20 + TCEP)	45	53	105
ED-20 + TCEP + Pristine WS_2_	51	71	138
ED-20 + TCEP + WS_2_ (5.0 wt% aminoacetic acid)	49	75	147
ED-20 + TCEP + WS_2_ (7.5 wt% aminoacetic acid)	43	66	158
ED-20 + TCEP + WS_2_ (10.0 wt% aminoacetic acid)	38	57	175

^1^ All systems were cured using 15 phr of PEPA hardener. ^2^ T_max_—maximum self-heating temperature during curing.

**Table 3:** Thermal curing parameters of epoxy compositions with pristine and aminoacetic acid-functionalized WS_2_ determined by differential scanning calorimetry (DSC).

Epoxy Composition ^1^	Curing Temperature Range (T_b_–T_e_) ^2^, °C	T_max hr_ ^3^, °C	Curing Enthalpy, J/g
ED-20 + TCEP + Pristine WS_2_	51–161	102	566
ED-20 + TCEP + WS_2_ (2.5 wt% aminoacetic acid)	46–162	101	613
ED-20 + TCEP + WS_2_ (5.0 wt% aminoacetic acid)	44–159	102	615
ED-20 + TCEP + WS_2_ (7.5 wt% aminoacetic acid)	43–161	102	639

^1^ All systems were cured using 15 phr of PEPA hardener. ^2^ T_b_—onset temperature of curing; T_e_—end temperature of curing. ^3^ T_max hr_—temperature at maximum heat release.

**Table 4:** Mechanical properties of epoxy nanocomposites modified with pristine and aminoacetic acid-functionalized WS_2_ nanoparticles (phr—parts per hundred resin). Values are presented as means ± standard deviations (n = 5).

Epoxy Composition ^1^	Flexural Strength, MPa	Flexural Modulus, MPa	Tensile Strength, MPa	Tensile Modulus, MPa	Impact Toughness, kJ/m^2^
ED-20 + TCEP	53 ± 1.8	1750 ± 50	36 ± 1.8	1610 ± 48	8.0 ± 0.32
+0.04 phr WS_2_	122 ± 3.6	3108 ± 92	58 ± 2.6	2246 ± 86	13.4 ± 0.53
+0.05 phr WS_2_	128 ± 3.8	3444 ± 98	62 ± 2.9	2568 ± 94	13.6 ± 0.54
+0.10 phr WS_2_	130 ± 3.9	3721 ± 105	62 ± 2.9	2601 ± 96	14.5 ± 0.56
+0.50 phr WS_2_	144 ± 4.1	4102 ± 115	66 ± 3.1	2690 ± 98	16.0 ± 0.61
+1.00 phr WS_2_	139 ± 4.0	4293 ± 120	53 ± 2.3	2758 ± 105	13.2 ± 0.52
+5.00 phr WS2	132 ± 3.9	4588 ± 127	41 ± 2.0	2917 ± 110	11.0 ± 0.44
+0.50 phr WS_2_—aminoacetic acid	168 ± 5.1	5282 ± 152	82 ± 2.5	3206 ± 125	17.0 ± 0.70
Statistical significance of differences (*p*-values)					
Control vs. WS_2_—aminoacetic acid ^2^ (0.5 phr)	<0.0001	<0.0001	<0.0001	<0.0001	<0.0001
WS_2_ (0.5 phr) vs. WS_2_—aminoacetic acid	<0.01	<0.01	<0.01	<0.01	~0.08 (n.s.) ^3^

^1^ All systems were cured using 15 phr of PEPA hardener. ^2^ WS_2_—aminoacetic acid refers to WS_2_ functionalized with 7.5 wt% aminoacetic acid. ^3^ n.s.—not statistically significant.

**Table 5:** Comparative analysis of WS_2_ nanoparticle surface modification methods in relation to chemical mechanisms, environmental considerations, dispersion effects, and epoxy compatibility.

Method	Mechanism of Interaction	Process Complexity	Environmental Friendliness	Effect on Dispersion	Chemical Bonding with Epoxy	Limitations
Silane Coupling Agents [31]	Si–O–WS_2_ covalent bonds via hydrolysis and condensation	Multistep; sensitive to moisture	Moderate (organic solvents required)	Improved (via silanol groups)	Moderate	Moisture sensitivity; residual byproducts; costly reagents
Polymer Grafting [32]	Grafting of reactive polymer chains onto WS_2_ surface	High; multistep synthesis	Low (synthetic monomers)	Excellent	Strong	Time-consuming; grafting control issues; high cost
Tannic Acid Modification [33]	Hydrogen bonding and π–π interactions	Simple; aqueous medium	High (bio-based, non-toxic)	Moderate	Weak (non-covalent)	Thermal instability; weak matrix interaction
Organophosphonate Treatment [34]	Polar interaction; possible covalent anchoring	Moderate	Moderate (some toxicity concerns)	Good	Moderate	High loading required; costly
Aminoacetic Acid (This Study)	Covalent esterification (–COOH with WS_2_); epoxy ring opening (–NH_2_ with matrix)	One-step; aqueous; mild conditions	High (green, non-toxic)	Excellent (dual reactive sites)	Strong (dual-site covalent bonding)	Requires optimization to avoid oversaturation

## Data Availability

The original contributions presented in this study are included in the article. Further inquiries can be directed to the corresponding author.

## Abbreviations

The following abbreviations are used in this manuscript:

2D	Two-dimensional
3D	Three-dimensional
BET	Brunauer–Emmet–Teller method
DGEBA	Diglycidyl ether of bisphenol A
DSC	Differential scanning calorimetry
EDS	Energy-dispersive X-ray Spectroscopy
FTIR	Fourier Transform Infrared Spectroscopy
PEPA	Polyethylene polyamine
phr	Parts by hundred resin
rpm	Revolutions per minute
SEM	Scanning Electron Microscopy
TCEP	Tris(2-chloroethyl) phosphate
TGA	Thermogravimetric analysis
TMDs	Transition metal dichalcogenides
XRD	X-ray diffraction
